# Preoperative γ-glutamyl transpeptidase to platelet ratio (GPR) is an independent prognostic factor for HBV-related hepatocellular carcinoma after curative hepatic resection

**DOI:** 10.1097/MD.0000000000004087

**Published:** 2016-07-08

**Authors:** Wan-Li Wang, Xing-Long Zheng, Zhi-Yong Zhang, Ying Zhou, Jie Hao, Gang Tang, Ou Li, Jun-Xi Xiang, Zheng Wu, Bo Wang

**Affiliations:** a Department of Hepatobiliary Surgery, First Affiliated Hospital, Health Science Center, Xi’an Jiaotong University, Xi’an; b Department of General Surgery, Bazhong Central Hospital, Bazhong, People's Republic of China.

**Keywords:** γ-glutamyl transpeptidase to platelet ratio, GPR, HCC, hepatocellular carcinoma, prognosis

## Abstract

Liver fibrosis and cirrhosis is associated with the prognosis of patients with hepatocellular carcinoma (HCC) after treatment. The γ-glutamyl transpeptidase to platelet ratio (GPR) is reported to predict significant liver fibrosis and cirrhosis. The aim of this study was to investigate the predictive value of preoperative GPR on the recurrence and survival of patients with HCC who underwent curative hepatectomy.

A retrospective review of demographics, medical records, and prognosis of patients with hepatitis B virus (HBV)–related HCC was performed. Overall survival (OS) and disease-free survival (DFS) were evaluated using Kaplan–Meier method, and the log-rank test was used to analyze differences in recurrence and survival. Univariate and multivariate analyses were used for significance of prognostic factor.

A total of 357 patients with HBV-related HCC were included in this analysis. The preoperative GPR was associated with recurrence and survival rates, independent of HCC progression or tumor marker levels, in a multivariate analysis. OS was higher in patients with a GPR <0.84 versus ≥084 (5-year survival rates 58.6% vs. 38.5%; *P* < 0.001). DFS was also worse in patients with a GPR ≥0.84 than in those with GPR <0.84 (5-year recurrence rates 42.8% vs. 22.8%; *P* < 0.001).

GPR score of ≥0.84 represents a major risk factor for the poor prognosis for HBV-related HCC after hepatic resection, and GPR served as an independent predictive factor for HBV-related HCC OS.

## 1 Introduction

Hepatocellular carcinoma (HCC), one of the most common malignancies, is ranked the second most frequent cause of death from cancer and the fifth common cancer from global cancer statistics.^[1]^ China alone accounts for about 50% of the total number of new liver cancer cases and deaths.^[1]^ Most of the cases are associated with liver cirrhosis on account of chronic hepatitis B virus (HBV) infection, particularly in China.^[2]^ Patients with HCC can achieve a 5-year survival rate approximately 70% by liver transplantation, radiofrequency ablation (RFA), or curative hepatic resection in early tumor stage; nevertheless, patients with advanced HCC have median survival time <1 year.^[3,4]^ Long-term survival remains far from satisfactory owing to the high cancer recurrence.^[5]^

Curative hepatic resection is considered to be the most powerful curative therapy for patients with HCC when liver transplantation is not immediately accessible or not an option.^[3]^ After resection of HCC, prognosis is dependent on factors such as tumor size and differentiation, vascular invasion, and resection margin status.^[5]^ Most of these factors are determined only after surgery. Therefore, the widespread research for a potential effective hematological prognostic indicator that would be available before surgery and improved prognosis by allowing earlier intervention.^[6,7]^ Inflammation-based prognostic scores have been formed to predict cancer survival. Subsets of peripheral blood, including lymphocytes, monocytes, platelets (PLT), and neutrophil to lymphocyte ratio, have been found to be associated with prognosis of different cancers.^[8–10]^ Anti-PLT therapy has been found in the prevention of HBV-related HCC.^[11]^ Studies concerning PLT status and HCC diagnosis with postoperative complication and survival have been extensively described.

To date, α-fetoprotein (AFP) is an immensely studied tumor biomarker for HCC prognosis.^[7]^ A meta-analysis shows that the most powerful predictors of death in patients with cirrhosis and HCC are liver related and/or tumor related; especially, AFP was among the most robust predictors.^[12]^ To date, many tumor markers including AFP-L3, glypican-3, γ-glutamyl transferase (GGT), DCP, GP73, and osteopontin have also been evaluated as substitutes or complements for AFP in the diagnosis and prognosis of HCC.^[4,6,7,13]^ Moreover, AFP concentration rose in 11% to 47% of patients with liver cirrhosis and in 15% to 58% of patients with chronic hepatitis. Thus, reevaluating tumor biomarkers to find potential candidates to be included in the surveillance of HCC prognosis might be of great importance in clinical practice.

Liver function is associated with HCC treatment selection and treatment response.^[14]^ Liver function parameters could be transiently deteriorated in patients after hepatectomy, transcatheter arterial chemoembolization, or RFA.^[14,15]^ Kuroda et al evaluated the liver function parameter changes after percutaneous RFA and concluded that a Child–Pugh score of ≥9 represents a main risk factor for the aggravation of liver function through a retrospective study.^[15]^ Previous study presented preoperative aspartate aminotransferase to platelet ratio as an independent prognostic factor for HBV-induced HCC after curative hepatic resection.^[16]^ What is noteworthy is that a recent study reports that the γ-glutamyl transpeptidase to platelet ratio (GPR) has the power to predict liver cirrhosis and fibrosis in chronic HBV-infected patients in West Africa.^[17]^ Therefore, as the most common tests in clinics, the roles of liver function parameters in HCC prognosis should be evaluated.

Considering all the aforementioned concerns, the aim of this study was to reevaluate the prognostic value of liver function parameters, clinicopathological characteristics, and treatment regimen in HCC overall survival (OS) after hepatic resection.

## 2 Patients and methods

### 2.1 Patients

The medical records of all patients with HCC who were admitted to the First Affiliated Hospital, Health Science Center, Xi’an Jiaotong University, during 2006 to 2013 were retrospectively reviewed to confirm the relevant clinicopathological parameters. HCC diagnosis was based on radiologic criteria according to European Association for the Study of the Liver and was finally verified pathologically after surgery.^[18]^ All experiments in this study were approved by the Ethics Committee of Health Science Center of Xi’an Jiaotong University and informed consent was signed. All patients, after hepatectomy, were monitored for a period of 7.1 to 106.3 months with a median length of 62.6 months till the end of 2014 by ultrasonography and enhanced CT or MRI every 3 to 6 months. The study included only patients with HBV-related HCC who underwent curative hepatic resection, and the cases without death in the perioperative period. Patients who are younger than 18 years old, with non–HBV-induced HCC, underwent noncurative hepatic resection, died in the perioperative period, or received sorafenib treatment before the recurrence of HCC were excluded from the study.

### 2.2 Study design and methods

This is a retrospective, single-center study. The demographics, diagnoses, preoperative condition, tumor markers, blood routine test, liver function parameters, surgical procedures, and postoperative outcomes of patients were extracted. We derived patients’ follow-up data from a review of inpatient and outpatient medical records and from direct follow-up visits. Following data were recorded for further analysis: sex, age, etiology, date of HCC diagnosis, date of recurrence, date of death or last information, presence of cirrhosis, HBV DNA, ascites, main serological parameters (PLT, aspartate aminotransferase, alanine aminotransferase, total bilirubin [TBIL], direct bilirubin, albumin, alkaline phosphatase, γ-glutamyl transpeptidase [GGT], prothrombin time [PT], AFP, etc.), tumor characteristics (number of lesions, location of lesions, diameter of largest lesion, portal vein invasion, organ invasion, nodes status), and surgical procedures.

OS was defined as the time from HCC diagnosis to the death due to any cause. Disease-free survival (DFS) denotes the chances of staying free of disease after hepatic resection for patients with HCC. Tumor differentiation was graded according to Edmonson–Steiner stage. Child–Pugh score (sometimes the Child–Turcotte–Pugh score) was calculated based on the severity of hepatic encephalopathy, ascites, TBIL, albumin, and PT. Tumor characteristics and liver histology status including cirrhosis, tumor size, multicentric tumor, portal vein invasion, and organ invasion were diagnosed by imaging studies such as enhanced CT/MRI.

### 2.3 Statistical analysis

Statistical analysis was performed with SPSS software (version 19.0; SPSS Inc, Chicago, IL) for analysis. Fisher's exact test (in cases of low frequency) and univariate χ^2^ were implemented to discriminate discrete variables. And nonparametric statistical analyses were used for non-normally distributed data. Differences between groups with continuous variables were tested by Mann–Whitney *U* tests and categorical variables were detected by χ^2^ analysis. OS between different groups was compared by the Kaplan–Meier method and the log-rank test was used to estimate the difference in survival. Factors independently related to survival were tested by a Cox regression model adjusted for a propensity score. Only covariates significantly associated with outcomes at univariate analysis (2-sided *P* < 0.05) were included in the multivariate analysis model. Results were reported as hazard ratio (HR) adjusted for the propensity score in 4 strata with 95% confidence intervals (CIs). *P* < 0.05 was considered significant.

### 2.4 Propensity score analysis

As patients with HBV-related HCC were grouped based on GPR cutoff value without any randomized study design, in order to control the group selection bias, a propensity score, as an adjustment variable, was built. A logistic regression model was developed to estimate the individual propensity score for the 357 patients. The model included covariates that may have influences on both the grouping and the survival (Table 1). The model showed 68.2% of correctly classified patients. Based on the estimated propensity score, patients were allocated to 4 strata, with each stratum having 25% of the patients. A multivariate analysis of the entire population was adjusted for the propensity score in 4 strata.^[16]^

**Table 1:**
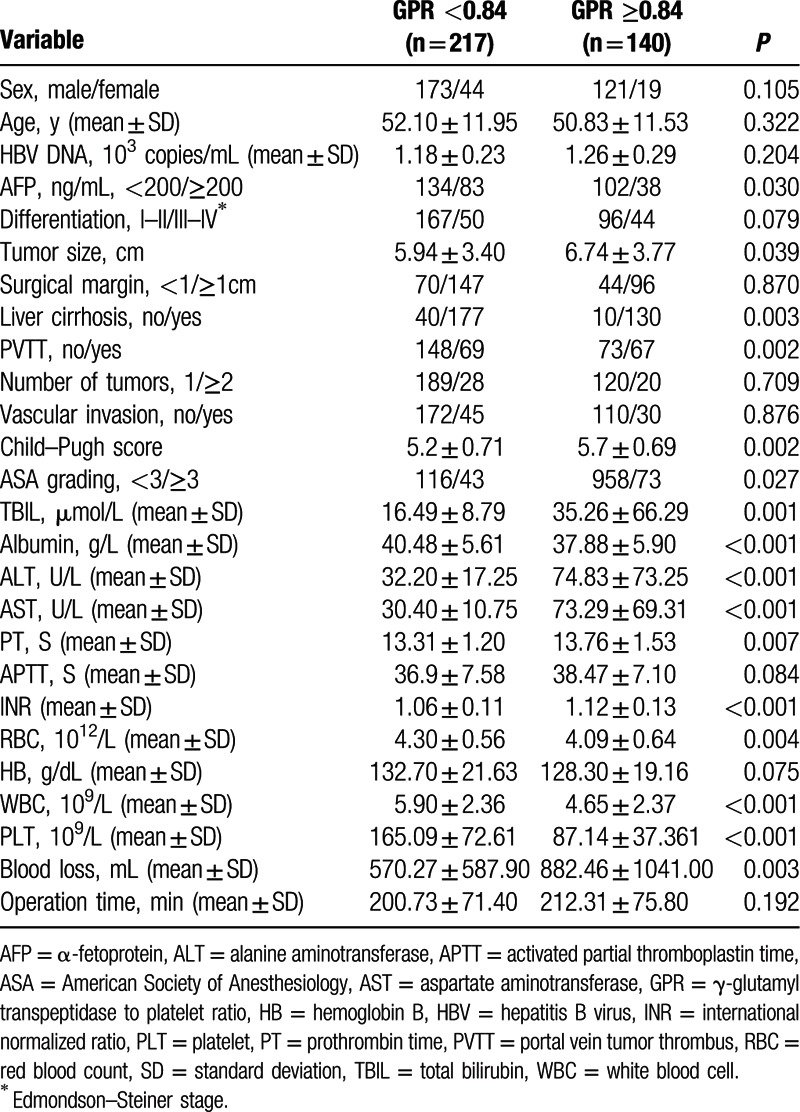
Baseline comparison between patients with GPR ≥0.84 and <0.84.

## 3 Results

### 3.1 Demographics and perioperative details

A total of 357 patients with HBV-related HCC who underwent curative hepatic resection were included in this study. There were 294 male patients (82.4%) and 63 female patients (17.6%). Recurrence occurred in a total of 220 patients (61.6%), whereas 170 patients (47.6%) died during follow-up. Hepatitis B surface antigen was positive in all patients, with 307 (86.0%) patients having underlying cirrhosis. Increased AFP (≥200 ng/mL) was found in 121 cases (33.9%), and 48 patients (13.4%) had multiple tumors. With regard to tumor differentiation according to Edmondson–Steiner stage, there were 263 (73.7%) stages I to II and 94 (26.3%) stages III to IV. Details of these features are shown in Table 1.

### 3.2 ROC curves of GPR for HCC overall survival

**Figure 1:**
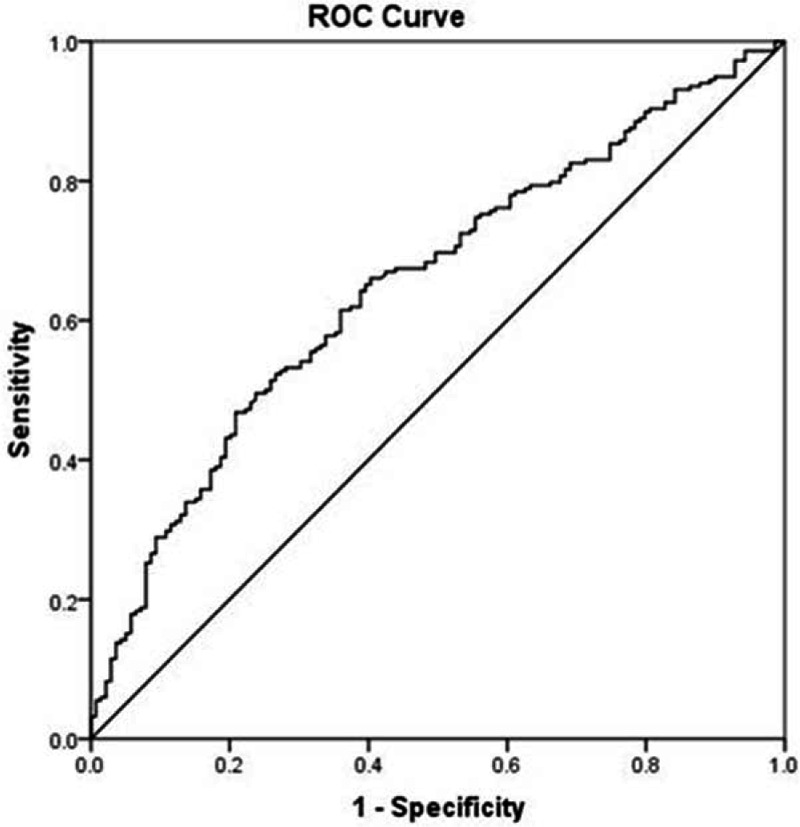
ROC curves for GPR in relation to death. AUROC curves were 0.653 (95% confidence interval, 0.596–0.710, *P* < 0.001) for GPR. The calculated cutoff value for GPR was 0.84, with sensitivity of 64.2% and specificity of 61.2%. AUROC = area under ROC curve, GPR = γ-glutamyl transpeptidase to platelet ratio, ROC = receiver operating characteristic.

To evaluate the predictive accuracy of serum GPR for HCC OS, we performed receiver operating characteristic (ROC) curve analyses indicating that elevated GPR could significantly more accurately predict HCC OS. An optimal cutoff value of 0.84 corresponded to the maximum joint sensitivity and specificity on the ROC plot for GPR (area under ROC = 0.653, 95% CI = 0.596–0.710, *P* < 0.001; Fig. 1), with sensitivity of 64.2% and specificity of 61.2% in the prediction of death.

### 3.3 Risk factors for prognosis of HCC after hepatectomy

Univariate analysis showed that AFP, Edmondson–Steiner stage, tumor size, surgical margin, portal vein tumor thrombus (PVTT), vascular invasion, GPR, blood loss, and blood transfusion were all factors related to the OS in patients with HCC (all *P* < 0.05). When these factors were evaluated by a multivariate model using forward selection, AFP, PVTT, GPR, tumor size, and blood transfusion were significantly associated with HCC OS (HR = 1.991, 95% CI = 1.179–3.178, *P* = 0.002; HR = 2.173, 95% CI = 1.471–3.198, *P* < 0.001; HR = 2.734, 95% CI = 1.681–3.832, *P* < 0.001; HR = 1.785, 95% CI = 1.178–3.426, *P* = 0.039; and HR = 1.734, 95% CI = 1.041–3.829, *P* = 0.015, respectively) (Table 2).

**Table 2:**
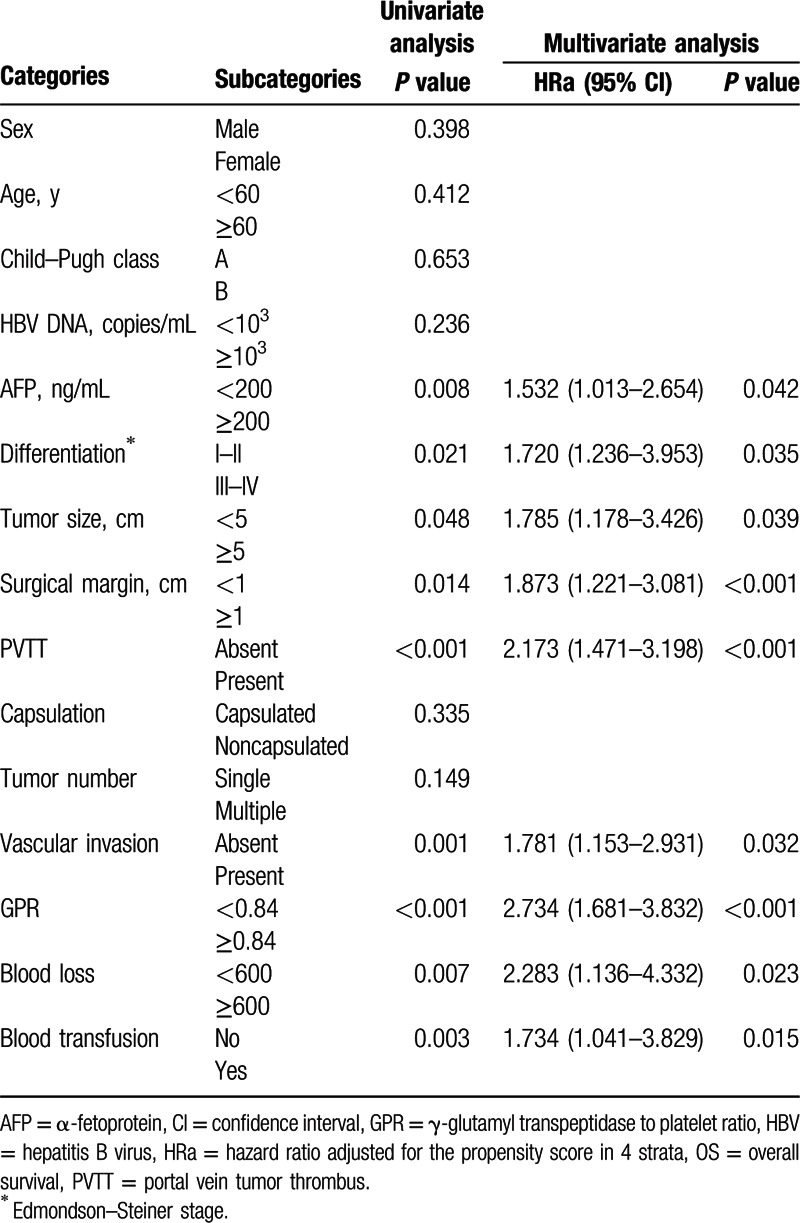
Univariate and multivariate analyses of OS after propensity score adjustment.

Univariate analysis identified AFP, tumor size, Edmondson–Steiner stage, surgical margin, PVTT, vascular invasion, GPR, blood loss, and blood transfusion as elements that have strong association with the DFS after patients’ hepatectomy. Based on the multivariate analysis, surgical margin, vascular invasion, GPR, and blood transfusion are also independent variables linked to lower DFS rates (Table 3).

**Table 3:**
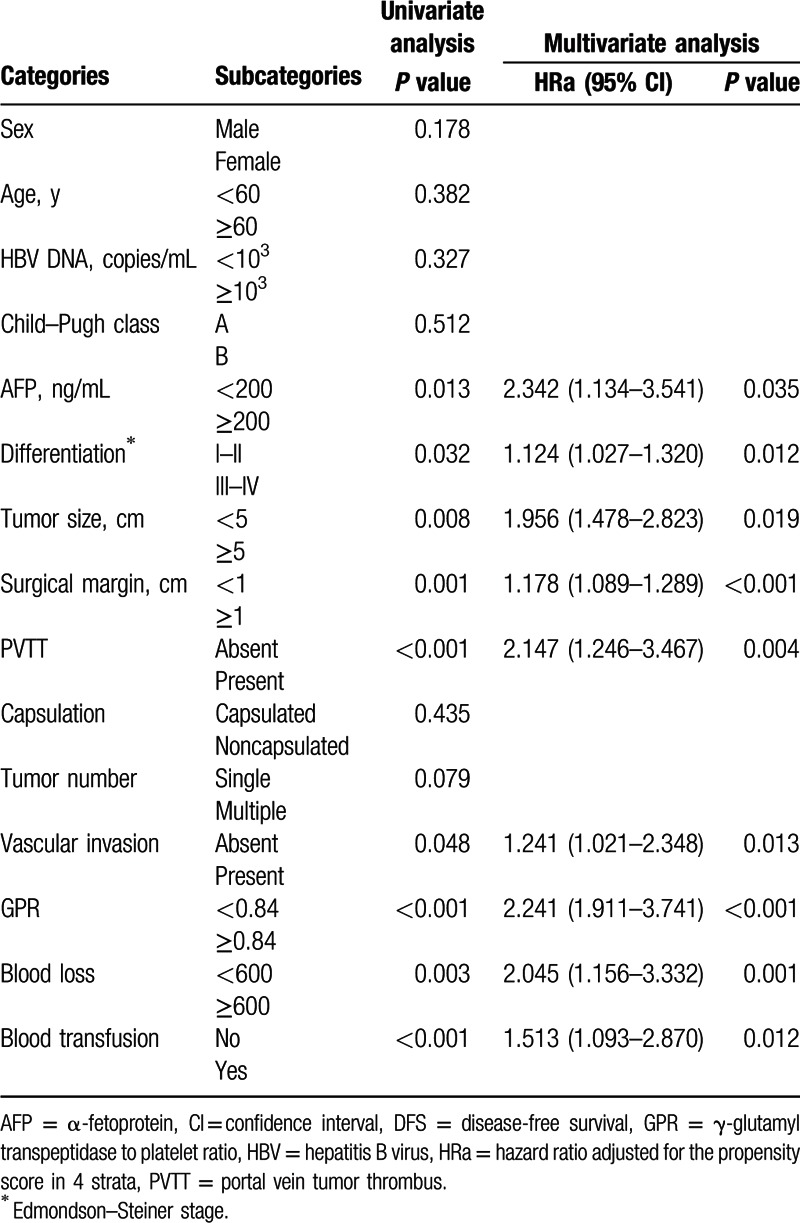
Univariate and multivariate analyses of DFS after propensity score adjustment.

**Figure 2:**
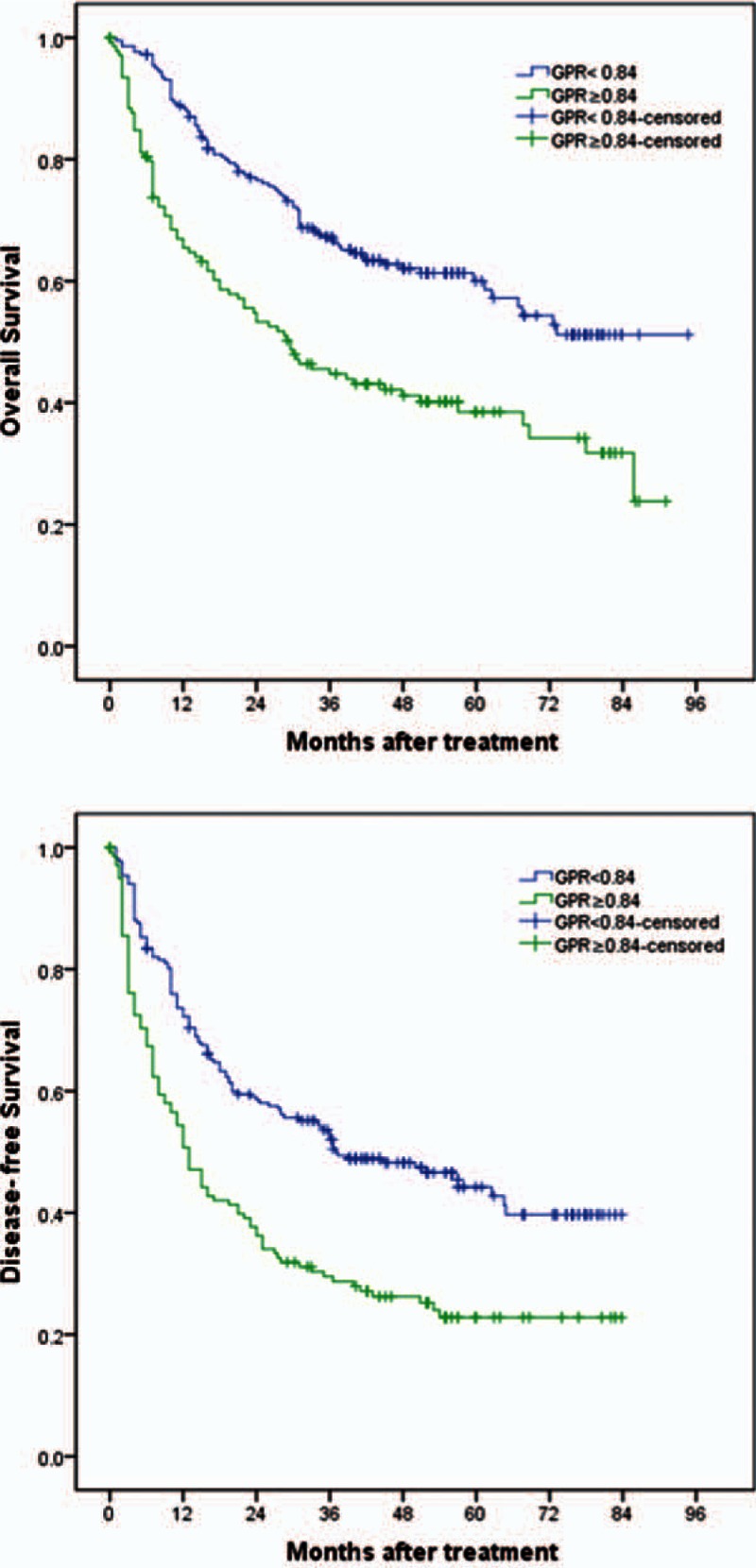
Kaplan–Meier survival plots comparing OS and DFS for patients stratified as low and high GPR groups. (A) OS of patients with GPR ≥0.84 was lower than in those with GPR <0.84 (*P* < 0.001, log-rank test); (B) DFS of patients with GPR ≥0.84 was worse than in those with GPR <0.84 (*P* < 0.001, log-rank test). DFS = disease-free survival, GPR = γ-glutamyl transpeptidase to platelet ratio, OS = overall survival.

As shown in Fig. 2, we performed a Kaplan–Meier event analysis. We grouped the GPR levels with abnormal cutoff values into normal group and elevated group. This revealed that the higher the levels of GPR, the greater is the risk of worse prognosis (all log rank *P* < 0.001). OS was higher in patients with a GPR <0.84 versus ≥0.84 (5-year survival rates 58.6% vs. 38.5%; *P* < 0.001). DFS was also worse in patients with a GPR ≥0.84 than in those with GPR <0.84 (5-year recurrence rates 42.8% vs. 22.8%; *P* < 0.001).

### 3.4 Relationship between clinicopathological features and GPR status

Clinicopathological features of patients with different GPR status are summarized in Table 1. AFP, tumor size, liver cirrhosis, PVTT, number of tumors, Child–Pugh score, American Society of Anesthesiology grading, TBIL, albumin, alanine aminotransferase, aspartate aminotransferase, PT, international normalized ratio, red blood cell, PLT, white blood cell, and blood loss were significantly different between the 2 groups (*P* < 0.05).

## 4 Discussion

HCC is one of the major health burdens in the world. To date, many tumor markers have been proposed in the early detection, diagnosis, and prognosis of HCC. Unfortunately, few tumor markers have been externally validated in HCC survival prediction. AFP is still the most widely studied and robust predictor of biomarker for HCC prognostication. Our previous studies have confirmed that AFP-L3% and osteopontin have significant prognostic value in patients with HCC.^[6,7]^ Furthermore, p53, glypican-3, GGT, DCP, and GP73 are also being evaluated as a complement or substitute for AFP in the diagnosis and prognosis of HCC, especially with low AFP concentration.^[4,19]^ In most cases, especially in Asia, it is associated with concomitant liver cirrhosis due to chronic HBV infection.^[1,20]^ Therefore, reevaluation of tumor biomarkers based on liver function tests and other clinicopathological features to find potential candidates to be included in the surveillance of HCC prognosis is of great importance in clinical practice.

Child–Pugh class based on liver function is one of the significant factors influencing the prognosis of patients with HCC.^[14,15]^ But the percentage of patients diagnosed with initial HCC with good liver function continues to increase, owing to improved surveillance methods and effective anti-HBV drug treatment. Child–Pugh class A liver function was found in the majority of patients with HCC who had curative hepatic resection. Hence, discriminating the prognosis of patients with HCC treated with curative hepatectomy regarding Child–Pugh class based on liver function is ineffective. Thrombocytopenia is a cirrhosis surrogate that is associated with HCC development.^[21]^ It has been found that anti-PLT therapy is effective in the prevention of HBV-induced HCC.^[11]^ Recently, a study reported a noteworthy finding that GPR has the power to predict liver cirrhosis and fibrosis in chronic HBV-infected patients in West Africa.^[17]^ In this study, we tried to associate PLT with liver function as a predictive variable, using a hematological parameter, the GGT to PLT ratio, or GPR for short, for the outcome of patients with HBV-related HCC who underwent curative hepatic resection. We found that levels of AFP, GPR, blood loss, and blood transfusion were statistically higher in patients with death outcome according to the baseline characteristics analysis of patients with HCC with different outcomes and more patients suffered from liver cirrhosis, PVTT, and vascular invasion in HCC death group. Because PVTT, portal vein invasion, and advanced Barcelona Clinic Liver Cancer staging have been considered to be associated with poor prognosis of patients with HCC,^[22,23]^ we assume that liver function parameters associated with PLT could be potential biomarkers for the prediction of HCC prognosis. In this study, we implemented further analysis of the relationship between these factors and HCC OS.

GGT has been regarded as a significant diagnostic marker for liver diseases.^[24]^ Hepatocarcinogenesis models showed that GGT expression was related to tumor formation and progression.^[24]^ Additionally, the GGT level was significantly higher in patients with HCC with larger tumors.^[21]^ Previous studies have pointed out that patients with elevated GGT level have a worse prognosis than those with low GGT level; therefore, GGT may be a potential tumor prognosis marker in HCC, especially in low-AFP cohort.^[25,26]^ A study carried out by Ruhl and Everhart has shown that elevated GGT was associated with mortality from all causes, diabetes, liver disease, and cancers.^[27]^ Another report from Ma et al showed that serum GGT is a convenient prognostic biomarker related to OS and DFS in patients with HCC treated by RFA.^[28]^ In addition to this, a study from Song et al also suggested that high levels of GGT and indocyanine green retention rate at 15 minutes (ICG-R15) can be preoperative predictor factor when evaluating postoperative tumor recurrence.^[29]^ In patients with chronic HBV infection, GGT has been previously considered as an independent variable for significant fibrosis^[30,31]^ and GPR can predict severe liver cirrhosis and fibrosis in chronic HBV-infected patients in West Africa.^[17]^ In most Asian cases, it is related to concomitant liver cirrhosis due to chronic HBV infection.^[2]^ Consistently with the previous studies, we performed ROC curve analyses of GPR for HCC OS and demonstrated that the GPR score of ≥0.84 represents a major risk factor for the adverse prognosis after hepatic resection. OS was higher in patients with a GPR <0.84 versus ≥0.84 (5-year survival rates 58.6% vs. 38.5%; *P* < 0.001). DFS was also worse in patients with a GPR ≥0.84 than in those with GPR <0.84 (5-year recurrence rates 42.8% vs. 22.8%; *P* < 0.001). The present study demonstrated that GPR can be an independent predictive factor for HBV-related HCC OS.

Although we can draw a conclusion that GPR served as an independent predictive factor for HBV-related HCC OS, there are several limitations in this study. First, this was a retrospective single-center study. Second, all patients with HCC were ethnically Chinese. Third, HBV infection was the only cause of liver fibrosis in all the patients. In order to extend our findings for general cases, multicenter clinical studies are needed, and patients of other ethnicities with various etiologies of liver fibrosis should be also evaluated.

## 5 Conclusions

In conclusion, serum GGT, among liver function tests, together with PLT should be a potential predictive factor for HCC OS. GPR score of ≥0.84 represents a major risk factor for the poor prognosis for HBV-related HCC after curative hepatic resection, and GPR served as an independent predictive factor for HBV-related HCC OS.

## Acknowledgment

The authors gratefully acknowledge Xian-ming Xu from Dalhousie University for polishing the article.

## References

[1] Torre LA Bray F Siegel RL Global cancer statistics, 2012. *CA Cancer J Clin* 2015; 65:87–108. 25651787 10.3322/caac.21262

[2] Cucchetti A Cescon M Trevisani F Current concepts in hepatic resection for hepatocellular carcinoma in cirrhotic patients. *World J Gastroenterol* 2012; 18:6398–6408. 23197885 10.3748/wjg.v18.i44.6398 PMC3508634

[3] Reddy SK Barbas AS Turley RS Major liver resection in elderly patients: a multi-institutional analysis. *J Am Coll Surg* 2011; 212:787–795. 21435922 10.1016/j.jamcollsurg.2010.12.048

[4] Rich N Singal AG Hepatocellular carcinoma tumour markers: current role and expectations. Best practice & research. *Clin Gastroenterol* 2014; 28:843–853. 10.1016/j.bpg.2014.07.018 25260312

[5] Forner A Llovet JM Bruix J Hepatocellular carcinoma. *Lancet* 2012; 379:1245–1255. 22353262 10.1016/S0140-6736(11)61347-0 PMC13537732

[6] Cheng J Wang W Sun C Meta-analysis of the prognostic and diagnostic significance of serum/plasma osteopontin in hepatocellular carcinoma. *J Clin Gastroenterol* 2014; 48:806–814. 24247813 10.1097/MCG.0000000000000018

[7] Cheng J Wang W Zhang Y Prognostic role of pre-treatment serum AFP-L3% in hepatocellular carcinoma: systematic review and meta-analysis. *PLoS One* 2014; 9:e87011. 24498011 10.1371/journal.pone.0087011 PMC3907387

[8] Sasaki A Iwashita Y Shibata K Prognostic value of preoperative peripheral blood monocyte count in patients with hepatocellular carcinoma. *Surgery* 2006; 139:755–764. 16782430 10.1016/j.surg.2005.10.009

[9] Liao R Tang ZW Li DW Preoperative neutrophil-to-lymphocyte ratio predicts recurrence of patients with single-nodule small hepatocellular carcinoma following curative resection: a retrospective report. *World J Surg Oncol* 2015; 13:265. 26328917 10.1186/s12957-015-0670-y PMC4557750

[10] Li MX Liu XM Zhang XF Prognostic role of neutrophil-to-lymphocyte ratio in colorectal cancer: a systematic review and meta-analysis. *Int J Cancer* 2014; 134:2403–2413. 24122750 10.1002/ijc.28536

[11] Sitia G Iannacone M Guidotti LG Anti-platelet therapy in the prevention of hepatitis B virus-associated hepatocellular carcinoma. *J Hepatol* 2013; 59:1135–1138. 23742914 10.1016/j.jhep.2013.05.040

[12] Tandon P Garcia-Tsao G Prognostic indicators in hepatocellular carcinoma: a systematic review of 72 studies. *Liver Int* 2009; 29:502–510. 19141028 10.1111/j.1478-3231.2008.01957.x PMC2711257

[13] Yang Z Ye P Xu Q Elevation of serum GGT and LDH levels, together with higher BCLC staging are associated with poor overall survival from hepatocellular carcinoma: a retrospective analysis. *Discov Med* 2015; 19:409–418. 26175398

[14] Li JX Wu H Huang JW The influence on liver function after transcatheter arterial chemoembolization combined with percutaneous radiofrequency ablation in patients with hepatocellular carcinoma. *J Formos Med Assoc* 2012; 111:510–515. 23021508 10.1016/j.jfma.2011.05.016

[15] Kuroda H Kasai K Kakisaka K Changes in liver function parameters after percutaneous radiofrequency ablation therapy in patients with hepatocellular carcinoma. *Hepatol Res* 2010; 40:550–554. 20546330 10.1111/j.1872-034X.2009.00613.x

[16] Shen SL Fu SJ Chen B Preoperative aspartate aminotransferase to platelet ratio is an independent prognostic factor for hepatitis B-induced hepatocellular carcinoma after hepatic resection. *Ann Surg Oncol* 2014; 21:3802–3809. 24849520 10.1245/s10434-014-3771-x

[17] Lemoine M Shimakawa Y Nayagam S The gamma-glutamyl transpeptidase to platelet ratio (GPR) predicts significant liver fibrosis and cirrhosis in patients with chronic HBV infection in West Africa. *Gut* 2015; [Epub ahead of print]. 10.1136/gutjnl-2015-309260 PMC4975834 26109530

[18] Bruix J Llovet JM Prognostic prediction and treatment strategy in hepatocellular carcinoma. *Hepatology* 2002; 35:519–524. 11870363 10.1053/jhep.2002.32089

[19] Liu J Ma Q Zhang M Alterations of TP53 are associated with a poor outcome for patients with hepatocellular carcinoma: evidence from a systematic review and meta-analysis. *Eur J Cancer* 2012; 48:2328–2338. 22459764 10.1016/j.ejca.2012.03.001 PMC3395767

[20] Ferlay J Shin HR Bray F Estimates of worldwide burden of cancer in 2008: GLOBOCAN 2008. *Int J Cancer* 2010; 127:2893–2917. 21351269 10.1002/ijc.25516

[21] Carr BI Guerra V Hepatocellular carcinoma size: platelets, gamma-glutamyl transpeptidase, and alkaline phosphatase. *Oncology* 2013; 85:153–159. 23988857 10.1159/000354416 PMC3863783

[22] Yang Z Zhuang L Szatmary P Upregulation of heat shock proteins (HSPA12A, HSP90B1, HSPA4, HSPA5 and HSPA6) in tumour tissues is associated with poor outcomes from HBV-related early-stage hepatocellular carcinoma. *Int J Med Sci* 2015; 12:256–263. 25798051 10.7150/ijms.10735 PMC4366630

[23] Shirabe K Kajiyama K Harimoto N Prognosis of hepatocellular carcinoma accompanied by microscopic portal vein invasion. *World J Gastroenterol* 2009; 15:2632–2637. 19496194 10.3748/wjg.15.2632 PMC2691495

[24] Hanigan MH Gamma-glutamyl transpeptidase: redox regulation and drug resistance. *Adv Cancer Res* 2014; 122:103–141. 24974180 10.1016/B978-0-12-420117-0.00003-7 PMC4388159

[25] Carr BI Pancoska P Branch RA Significance of increased serum GGTP levels in HCC patients. *Hepatogastroenterology* 2010; 57:869–874. 21033244

[26] Carr BI Guerra V Giannini EG Low alpha-fetoprotein HCC and the role of GGTP. *Int J Biol Markers* 2014; 29:e395–402. 24832180 10.5301/jbm.5000092

[27] Ruhl CE Everhart JE Elevated serum alanine aminotransferase and gamma-glutamyltransferase and mortality in the United States population. *Gastroenterology* 2009; 136:477–485.e411. 19100265 10.1053/j.gastro.2008.10.052

[28] Ma H Zhang L Tang B Gamma-glutamyltranspeptidase is a prognostic marker of survival and recurrence in radiofrequency-ablation treatment of hepatocellular carcinoma. *Ann Surg Oncol* 2014; 21:3084–3089. 24748164 10.1245/s10434-014-3724-4

[29] Song P Inagaki Y Wang Z High levels of gamma-glutamyl transferase and indocyanine green retention rate at 15 min as preoperative predictors of tumor recurrence in patients with hepatocellular carcinoma. *Medicine* 2015; 94:e810. 26020384 10.1097/MD.0000000000000810 PMC4616400

[30] Myers RP Tainturier MH Ratziu V Prediction of liver histological lesions with biochemical markers in patients with chronic hepatitis B. *J Hepatol* 2003; 39:222–230. 12873819 10.1016/s0168-8278(03)00171-5

[31] Eminler AT Irak K Ayyildiz T The relation between liver histopathology and GGT levels in viral hepatitis: more important in hepatitis B. *Turk J Gastroenterol* 2014; 25:411–415. 25254524 10.5152/tjg.2014.3693

